# QPath: a method for querying pathways in a protein-protein interaction network

**DOI:** 10.1186/1471-2105-7-199

**Published:** 2006-04-10

**Authors:** Tomer Shlomi, Daniel Segal, Eytan Ruppin, Roded Sharan

**Affiliations:** 1 School of Computer Science, Tel-Aviv University, Tel-Aviv 69978, Israel; 2 Dept. of Molecular Microbiology and Biotechnology, Tel-Aviv University, Tel-Aviv 69978, Israel; 3 School of Medicine, Tel-Aviv University, Tel-Aviv, Tel-Aviv 69978, Israel

## Abstract

**Background:**

Sequence comparison is one of the most prominent tools in biological research, and is instrumental in studying gene function and evolution. The rapid development of high-throughput technologies for measuring protein interactions calls for extending this fundamental operation to the level of pathways in protein networks.

**Results:**

We present a comprehensive framework for protein network searches using pathway queries. Given a linear query pathway and a network of interest, our algorithm, QPath, efficiently searches the network for homologous pathways, allowing both insertions and deletions of proteins in the identified pathways. Matched pathways are automatically scored according to their variation from the query pathway in terms of the protein insertions and deletions they employ, the sequence similarity of their constituent proteins to the query proteins, and the reliability of their constituent interactions. We applied QPath to systematically infer protein pathways in fly using an extensive collection of 271 putative pathways from yeast. QPath identified 69 conserved pathways whose members were both functionally enriched and coherently expressed. The resulting pathways tended to preserve the function of the original query pathways, allowing us to derive a first annotated map of conserved protein pathways in fly.

**Conclusion:**

Pathway homology searches using QPath provide a powerful approach for identifying biologically significant pathways and inferring their function. The growing amounts of protein interactions in public databases underscore the importance of our network querying framework for mining protein network data.

## Background

Sequence homology searches have been the workhorse of bioinformatics for the past 30 years, providing the means to study the function and evolution of genes and proteins. Recent technological advances in large-scale measurements of protein-protein interactions (PPIs) such as yeast two-hybrid screens [1,2] and protein co-immunoprecipitation assays [3-5] have allowed us to shift our perspective from single genes and proteins to more complex functional units, such as protein pathways and complexes. Studying the function and evolution of protein modules underscores the importance of extending homology search tools from the single gene level to the network level.

In contrast to the vast research on gene and protein homology detection, there are only a few studies on homology detection at the network level, including studies on PPI networks [6-8], metabolic networks [9-12], and gene expression networks [13-16]. Most of these studies have focused on the identification of network regions that are conserved across several species. Initial attempts at the problem of query searches, i.e. searching for instances of a query subnetwork within a given network, have been made by Kelley et al. [6] and Pinter et al. [12] but both methods were limited in their applicability. The PathBLAST algorithm of Kelley et al. was designed to compare two protein networks and identify conserved pathways (linear, non-branching paths of interacting proteins). By constraining one of the networks to be a single pathway, PathBLAST was also applied for query searches. The use of the PathBLAST algorithm in this context has several drawbacks: (a) proteins may occur more than once in an identified matched pathway, which is biologically implausible; (b) the algorithm provides limited support for identifying non-exact pathway matches, supporting no more than a single consecutive deletion of proteins from the query pathway and no more than a single consecutive insertion of proteins to the matched pathway; and (c) the running time of the algorithm involves a factorial function of the pathway length, limiting its applicability to short pathways (in practice, it was applied to paths of up to 5 proteins). Pinter et al. have recently developed a pathway alignment tool called MetaPathwayHunter and applied it to mine metabolic networks. The algorithm enables fast queries of more general pathways that take the form of a tree (a subnetwork with no cycles). However, it is limited to searching within a collection of trees rather than within a general network. Finally, Leser has developed a query language for mining biological networks [17].

Here we give a novel comprehensive framework for querying linear pathways within a given network. Our algorithm, QPath, searches for matching pathways composed of distinct proteins that are similar to the query proteins in their sequence and interaction patterns. The matched pathways are scored according to their level of variation from the query pathway in terms of protein insertions and deletions, the sequence similarity of their constituent proteins to the query proteins, and the reliability of their constituent interactions. We provide a computational method for estimating the weight of each of these terms in the overall score, so as to maximize the fraction of the functionally significant matching pathways identified.

We applied QPath to analyze the PPI networks of the yeast *S. cerevisiae*, the fly *D. melanogaster*, and human, aiming to address two coupled, fundamental questions motivated from sequence analysis: (i) Can pathway homology be used to identify functionally significant pathways? (ii) Can one infer the function of a pathway based on pathway homology information? We provide positive answers to both questions. Notably, our finding that matched pathways in fly tend to preserve the function of their corresponding query pathways in yeast, has enabled us to derive a first annotated map of protein pathways in fly that are conserved from yeast.

## Results

### The QPath algorithm

**Figure 1:**
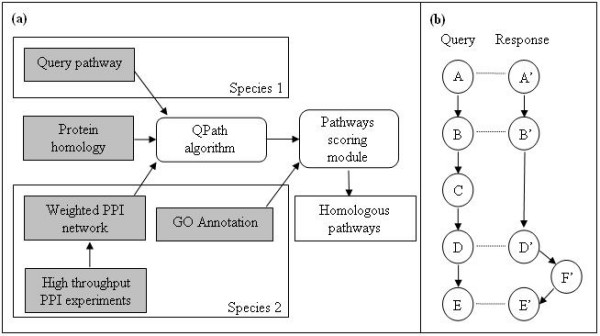
**The QPath algorithmic flow**. (a) Given a query pathway, a weighted PPI network, and sequence similarity scores between the query proteins and the network proteins, the QPath algorithm identifies a set of matching pathways. These are scored to capture the tendency of their constituent proteins to have a coherent function. (b) An example of an alignment that induces protein insertions (F') and deletions (C).

We developed a novel algorithm for querying a given protein network with a linear pathway of interest. The algorithm searches for matching pathways that are similar to the query in their sequence and interaction patterns. It relies on efficient graph-theoretic techniques, allowing it to process long pathways (up to 10 proteins) in minutes (see Methods and Supp.1 Table 3). While the algorithm can be applied to query any gene or protein network, we focus the discussion on its applications to mining PPI networks. QPath receives as input a query pathway consisting of a linear chain of interacting proteins; a PPI network with reliability scores for its interactions; and sequence similarity scores between the query proteins and the network proteins (Figure 1a). Similar to sequence alignment, the algorithm aligns the query pathway to putative pathways in the target network, so that proteins in analogous positions are sequence similar. Each matched pathway may contain a (bounded) number of protein insertions, representing proteins not aligned to the query proteins, and protein deletions, representing omission of matches to some query proteins (Figure 1b). The pathways are scored based on a *sequence score*, which measures their sequence similarity to the query pathway; an *interaction score*, which measures the reliability of their constituent interactions; and the number of protein insertions and deletions they employ. The top-scoring pathways are identified using a dynamic programming based algorithm that guarantees that matched pathways will be comprised of distinct proteins. The output of the algorithm is a set of non-redundant, significant matching pathways. The QPath program is available upon request.

### Pathway queries in the yeast and fly networks

To evaluate the utility of our algorithm in analyzing PPI networks, we applied it to the yeast and fly protein interaction networks, which are the largest and most well investigated networks in public databases [18]. As a first test of the algorithm, similarly to [6] , we queried the yeast network with the yeast filamentous growth MAPK cascade. The algorithm correctly recovered two known homologous MAPK pathways as the top matches (Supp. Figure 6). Next, we wished to perform a systematic evaluation of the algorithm's performance on the yeast and fly networks. Since the yeast network is supported by many more large-scale experiments [18] and, hence, expected to be more complete and accurate, we reasoned that by querying putative yeast pathways within the fly network we could reveal novel functional pathways therein, capitalizing on the more complete information in yeast.

To obtain a comprehensive set of putative pathways in the PPI network of yeast, we applied a modified version of the QPath algorithm to search the network for pathways that have high interaction scores (not based on specific query pathways, see Methods). The search was limited to pathways consisting of 6 proteins to achieve reasonable running times when applying QPath to query those pathways while allowing for (up to 3) insertions and deletions. We identified a set of 271 non-redundant pathways whose scores exceeded those of 99% of randomly chosen pathways (see Methods). The full list of identified pathways appears on the supplemental website [19].

We used two standard methods to assess the quality of these pathways (see Methods and Table 1): (i) Functional enrichment – representing the tendency of the pathway's proteins to have coherent Gene Ontology (GO) functions; and (ii) Expression coherency – measuring the similarity in expression profiles of the pathway's coding genes across different experimental conditions. In total, 80% of the yeast pathways were functionally enriched. In addition, the resulting pathways were significantly coherently expressed (Wilcoxon rank *p < 1e-300*). The significant functional enrichment and expression coherency of the identified pathways suggest that these pathways are biologically significant. In agreement with the expected lower quality of the fly network, we observed lower rates of functional enrichment and expression coherency when analyzing analogously-computed high-scoring pathways in fly (Table 1).

**Figure 2:**
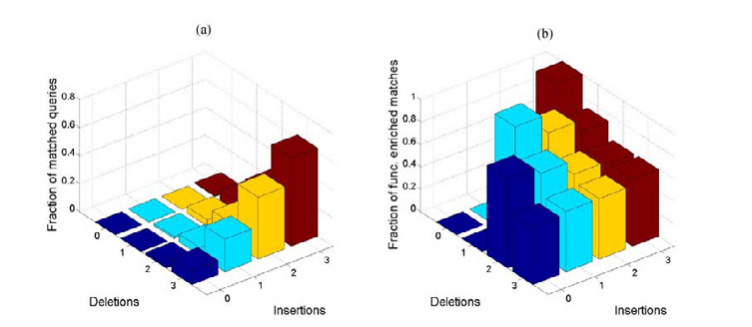
**Properties of matched pathways in different indel categories**. (a) The fraction of yeast queries with identifiable matching fly pathway out of all yeast queries within different indel categories. (b) The fraction of matched fly pathways that are functionally enriched out of all matched fly pathways in each indel category. Indel categories not covered by any matched pathway were marked as having 0% functionally enriched pathways.

For each significant pathway in yeast we executed the QPath algorithm to search for matching pathways in fly. In total, 63% of the yeast queries had matches in fly with up to three insertions and deletions. Given a yeast query, the probability of finding matching pathways in fly was highly correlated with the interaction score of the query (Spearman *p = 2.1e-04*). Only few of the queries had matching pathways with no insertions or deletions, implying that the algorithm's support for insertions and deletions was essential for identifying matching pathways (Figure 2a and Supp.1 Table 2a).

A query pathway potentially gives rise to multiple matching pathways, each with a different sequence score, interaction score and *indel category*, defined by the number of insertions and deletions employed by the pathway. In order to compare sequence and interaction scores for pathways from different indel categories, we normalized their scores by the number of proteins and interactions they contain, respectively. We found a statistically significant correlation between the functional enrichment of the matched pathways and their normalized interaction and sequence scores (Spearman *p *= 4e-15 and *p *= 0.003 for interaction and sequence scores, respectively). Furthermore, the indel category of a pathway was also found to be correlated with its functional enrichment: as expected, fly pathways exhibiting fewer protein insertions and deletions (hence, better conserving the query proteins) tended to be more functionally enriched than more distant pathway matches (Figure 2b and Supp.1 Table 2b).

Motivated by these observations, we devised a scoring scheme that assigns each pathway a score reflecting its estimated probability to be functionally enriched given its inherent characteristics, i.e., the number of insertions and deletions it employs and its normalized interaction and sequence scores (Methods). For each yeast query we refer to the matched pathway with the highest obtained score and, hence, most likely to be functionally enriched, as the *best-match *pathway.

**Figure 3:**
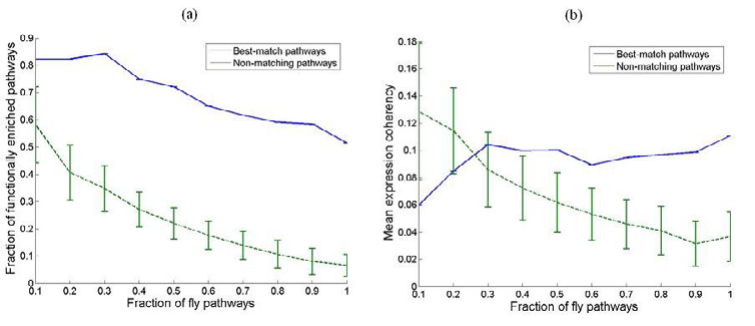
**Functional significance of best-match pathways infly **Functional enrichment (a) and expression coherency (b) of fly best-match pathways obtained by QPath compared to fly pathways that are not the result of a query. x-axis: Fraction of best-match pathways in fly. y-axis in (a): Fraction of functionally enriched pathways out of the set of pathways determined by x. y-axis in (b): Mean expression coherency of the pathways determined by x. The random pathway curves show the mean and standard deviation of the functional enrichment and expression coherency computed for random choices of pathway sets in fly.

To assess the biological significance of the best-match pathways in fly, we compared their functional enrichment and expression coherency to that of fly pathways that are not the results of a query. In total, 51% of the best-match pathways were functionally enriched. Within the set of 20% of the best-match pathways which were predicted to have the highest probability to be functionally enriched, 91% were indeed functionally enriched (Figure 3a). In comparison, the percentage of functionally enriched pathways in a set of fly pathways with the same length and distribution of interaction scores was 5%, which is significantly lower (*p < 1e-4*). The expression coherency of the best-match pathways was also significantly higher than that of randomly chosen pathways (*p < 1e-4*, Figure 3b). These results suggest that best-match pathways are biologically significant.

### Function conservation in yeast to fly pathways

Next, we investigated whether pathway similarity may be used to infer the function of a matched pathway based on the known function of the corresponding query pathway. Overall, out of the 171 yeast query pathways with an identified fly best-match pathway, 69 were functionally enriched and had a functionally enriched fly best-match pathway. Moreover, for 64% of these queries, the fly best-match pathways preserved one or more functions of the corresponding yeast query pathways. In contrast, when randomly shuffling the matches between fly pathways and yeast queries, only 31% of the fly pathways exhibited conservation of function (*p *< 1e-04). Interestingly, the pathway-based conservation of function was also much higher than the function conservation level among yeast-fly best sequence match proteins, which is estimated at 40% [6].

**Figure 4:**
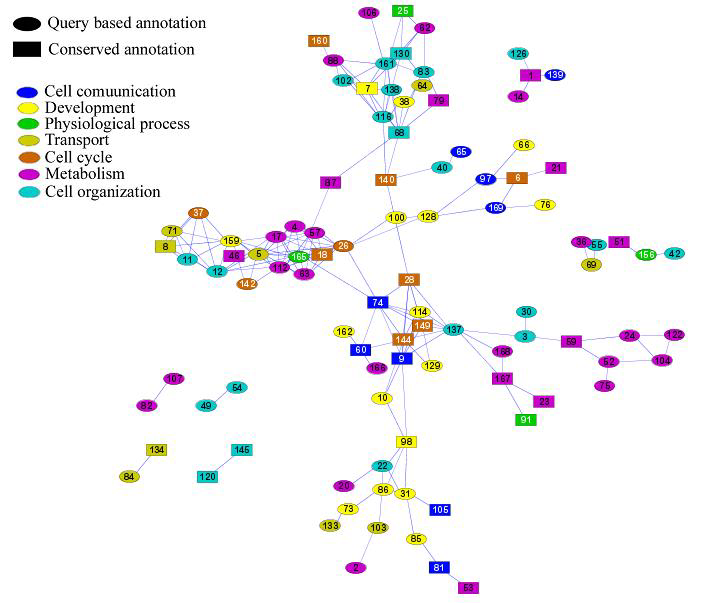
**Fly best-match pathway map**. A map of yeast best-match pathways in fly. Nodes represent best-match pathways and edges connect pathways that share at least two proteins. Each node is colored according to the enriched function of the corresponding query pathway in yeast. Pathways whose predicted annotation is also enriched among their constituent proteins appear as boxes; all other pathways appear as ellipses. Specific pathways can be looked up according to their number in the supplemental website [19].

We used the observed function conservation to derive a functional annotation of all fly best-match pathways, based on the enriched functions of their corresponding queries in yeast. Figure 4 summarizes these results in an annotated map of conserved fly (best-match) pathways. The map exhibits a modular structure, where groups of pathways overlap to define distinct network regions with common functions (the clustering coefficient is 0.26, significantly higher than in random networks that preserve vertex degrees (*p *< 0.05)). To evaluate the statistical significance of these predicted annotations, we computed for each best-match pathway the prevalence of the predicted annotation among its proteins (using a hypergeometric score), and compared these statistics with results obtained after randomizing the matches between yeast and fly pathways. The predicted annotations were found to be significantly more prevalent (*p *< 1e-04).

### Querying known signaling pathways from yeast and human

**Figure 5:**
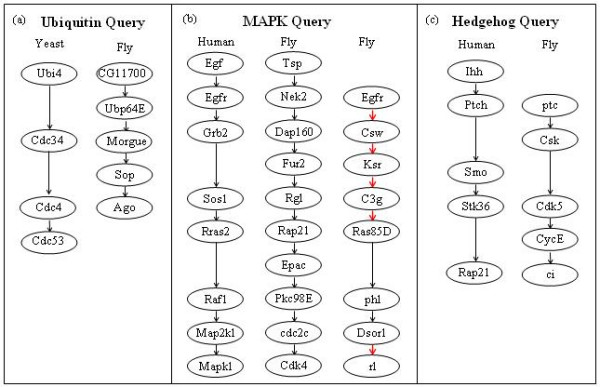
**Yeast and human queries and their best-matches in fly**. Yeast and human pathway queries and their best-matches in fly. (a) Yeast ubiquitin ligation pathway query in fly. (b) Human MAPK pathway query in fly. The pathway denoted by an asterisk is the result of querying a combined network of PPIs and genetic interactions (appearing in red). (c) Human Hedgehog pathway query in fly.

To demonstrate the use of our algorithm in a BLAST-like manner to query known protein pathways, we applied it to search the fly network for matches to queries consisting of known signaling pathways from yeast and human. As a first example, we used a ubiquitin-ligation pathway in yeast to query the fly network (Figure 5a). We identified a putatively homologous pathway in fly that is likely to be involved in protein degradation as well. Three out of its five proteins were annotated as being involved in ubiquitin-dependent protein degradation: Ubp64E is a putative ubiquitin-specific protease; morgue is annotated as a ubiquitin conjugating enzyme involved in apoptosis; and ago is a bona fide component of the SCF ubiquitin ligase complex [20,21]. Eye growth defects common to Ubp64E and ago mutants, may suggest that this pathway functions in the regulation of growth and apopotosis.

As a second example, we used two signaling pathways in human as queries to the fly network: a MAPK cascade and a Hedgehog signaling pathway. The top-scoring pathway in each case agreed well with the known functional annotations in fly. The MAPK query and its best-match are shown in Figure 5b. As expected for a MAPK-based signaling cascade, Nek2 is a putative receptor signaling protein serine/threonine kinase. Tsp is likely a growth factor, based on its EGF-like domain, which could serve as a ligand for Nek2. Dap160 and Fur2 are experimentally proven to be involved in receptor processing and internalization, respectively [22]. Although no experimental information is available for Rgl, Rap21, Epac and pkc98E, all available annotations fit into a G-protein coupled receptor protein signaling pathway: Rgl is a putative RAL GDP-dissociation stimulator, Rap21 has putative GTPase activity, Epac has putative cyclic nucleotide-dependent guanyl-nucleotide exchange factor activity, and both pkc98E and cdc2c are annotated as protein serine/threonine kinases. Interestingly, RNAi against cdc2c causes abnormal growth of cells in culture [23] , and the phenotype of mutant Nek2 implicates it in the regulation of mitosis [24]. Taken together, these evidences suggest that the inferred pathway could be involved in a cell-cell communication signaling cascade that regulates cell proliferation.

Figure 5c shows the fly pathway that best matches the human hedgehog signaling query. The known annotation of the pathway's proteins agrees well with its putative role in hedgehog signaling: ptc is a bona-fide receptor of hedgehog located at the plasma membrane [25]. Csk, annotated as a protein-tyrosine kinase, could well serve to further transmit the signal from ptc downstream. The cyclin-dependent protein kinase Cdk5, in association with the cyclin CycE, are well poised to further transmit the signal to the ultimate transcription factor ci. Ample experimental data show that ci, like ptc participates in the hedgehog signaling pathway, which in flies regulates cell growth in many tissues [25].

## Discussion and conclusion

We have presented a novel framework for querying linear pathways in PPI networks, allowing both deletions of proteins from the query pathway and insertions of proteins to the matched pathway. Matched pathways are assigned with scores reflecting their tendency to be functionally enriched, based on their variation from the query pathway, the sequence similarity of their proteins to the query proteins, and the reliability of their constituent interactions.

The effectiveness of the algorithm was demonstrated in querying the fly PPI network using protein pathways from yeast and human. When applying the algorithm to search for yeast pathway queries in fly, the matching pathways were significantly more functionally enriched compared to arbitrary pathways in the fly network. The resulting pathways tended to preserve the function of the original query pathways, demonstrating the applicability of our tool for predicting pathway function much in the same way as gene and protein functions are predicted using BLAST.

As with any PPI network study, it is important to deal with the vast amounts of noise present in the protein interaction data [26-28]. To handle false positive interactions we have assigned confidence scores to the interactions. To examine the contribution of the confidence scores for finding biologically-meaningful pathways, we repeated the functional enrichment and expression coherency analyses for sets of randomly chosen pathways from the yeast and fly networks obtained by discarding the interaction confidence scores. The percent of functionally enriched pathways and expression coherency rates found in these random sets were significantly lower than those found for high-scoring pathways (Table 1, Supp. Figure 7). Moreover, for both yeast and fly we found a statistically significant correlation between interaction scores and functional enrichment (Spearman correlation of 0.47 and 0.29, respectively, with *p < 1e*-300).

Accommodating for false negatives is a difficult challenge, but QPath handles those to some extent by allowing the introduction of protein indels to the matching pathway. Incorporating genetic interactions in the network may also help to tackle the problem of false negatives, as genetic interactions may indicate physical interactions between proteins [29]. In particular, for fly, the set of genetic interactions reported in FlyGRID [30] has significant overlap with the physical network, with a hyper-geometric *p*-value of *3.9e-7*. To test whether merging genetic and physical interactions contributes to the identification of functionally significant pathways, we applied QPath to re-query the human MAPK pathway in the merged network of fly (Figure 5b). The pathway identified is a variant of the EGFR receptor-kinase-signaling cascade, and five out of its seven proteins appear in the curated homologous fly pathway in KEGG [31]. The hypothetical signal is transmitted to the EGF receptor, and further relayed through ksr and C3G, a proven kinase and an annotated Ras guanyl-nucleotide exchange factor, respectively, to Ras85D. The latter has been shown experimentally to activate phl [32]. The putative signal is further transmitted to the MAP kinase kinase Dsor1, and downstream to rl, an annotated nuclear MAP kinase which likely activates specific transcription factors. Furthermore, ksr, phl, Dsor1 and rl are all required for modulation of the EGFR-mediated Ras85D mitogenic response [33]. Using genetic interactions is crucial for identifying this pathway as 5 out of its 7 interactions are genetic. This result suggests that merging both genetic and physical interactions may help coping with undetected protein-protein interactions.

We have only just begun to explore the world of protein networks, with the first drafts of the human PPI network just coming out [34,35]. With an ever increasing amount of genomes sequenced and protein interaction networks recovered, it is becoming increasingly important to develop tools for interpreting these data to provide detailed models of cellular machinery across organisms. We expect QPath to take a growing role in this exploration, giving essential means to use existing knowledge for inferring novel pathways and their function.

## Methods

### Data acquisition and processing

Protein-protein interaction data for yeast and fly were downloaded from DIP ([18] ; April 2005 download) and contained 15,166 interactions among 4,726 proteins in yeast, and 22,837 interactions among 7,028 proteins in fly (for fly, we complemented the DIP data by interactions from [36] ). Additional 2378 genetic interactions in fly were downloaded from FlyGRID [30]. To assign confidence scores to these interactions we used the logistic-regression-based scheme employed in [8]. Briefly, true positive and true negative interactions were used to train a logistic regression model, which assigns each interaction a reliability score based on the experimental evidence for this interaction, which includes the type of experiments in which the interaction was observed, and the number of observations in each experimental type. For yeast, we partitioned the experiments into four categories: co-immunoprecipitation screens [3,4] , yeast two-hybrid assays [2,37,38] , large scale experiments (other studies denoted as *exp:g *class in DIP) and small scale experiments (denoted as *exp:s *class in DIP). For fly, due to the smaller number of interaction screens available, we used each of three available large-scale screens [36,39,40] as a separate category. In addition, we used small scale fly experiments as a fourth category.

### Pathway alignment

We represent a PPI network using an undirected weighted graph *G *with a set *V *of *n *vertices, representing proteins, a set *E *of m edges, representing interactions, and an edge weight function *w(·,·) *representing interaction reliabilities. Given a pathway query *Q = (q_*1*_,...,q_*k*_)*, let *h*(*q*_*i*_*, j*) denote a sequence similarity score between query node *q*_*i *_and vertex *j *∈ *V*. An alignment of *Q *in *G *is defined as a pair *(P, M)*, where *P = (p_*1*_,...,p_*k*_) *is a matched path in *G*, and *M *is a mapping of query nodes onto *P *∪ *{0}*. The alignment allows up to *N*_*ins *_insertions and up to *N*_*del *_deletions, where deleted query nodes are mapped to *0 *by *M*. The weight of an alignment is a summation of the *interaction score*, ∑i=1l−1w(pi,pi+1)
 MathType@MTEF@5@5@+=feaafiart1ev1aaatCvAUfKttLearuWrP9MDH5MBPbIqV92AaeXatLxBI9gBaebbnrfifHhDYfgasaacH8akY=wiFfYdH8Gipec8Eeeu0xXdbba9frFj0=OqFfea0dXdd9vqai=hGuQ8kuc9pgc9s8qqaq=dirpe0xb9q8qiLsFr0=vr0=vr0dc8meaabaqaciaacaGaaeqabaqabeGadaaakeaadaaeWbqaaiabdEha3naabmaabaGaemiCaa3aaSbaaSqaaiabdMgaPbqabaGccqGGSaalcqWGWbaCdaWgaaWcbaGaemyAaKMaey4kaSIaeGymaedabeaaaOGaayjkaiaawMcaaaWcbaGaemyAaKMaeyypa0JaeGymaedabaGaemiBaWMaeyOeI0IaeGymaedaniabggHiLdaaaa@4123@ and the *sequence score*, ∑i=1,p′i≠0kh(qi,M(pi))
 MathType@MTEF@5@5@+=feaafiart1ev1aaatCvAUfKttLearuWrP9MDH5MBPbIqV92AaeXatLxBI9gBaebbnrfifHhDYfgasaacH8akY=wiFfYdH8Gipec8Eeeu0xXdbba9frFj0=OqFfea0dXdd9vqai=hGuQ8kuc9pgc9s8qqaq=dirpe0xb9q8qiLsFr0=vr0=vr0dc8meaabaqaciaacaGaaeqabaqabeGadaaakeaadaaeWbqaaiabdIgaOnaabmaabaGaemyCae3aaSbaaSqaaiabdMgaPbqabaGccqGGSaalcqWGnbqtdaqadaqaaiabdchaWnaaBaaaleaacqWGPbqAaeqaaaGccaGLOaGaayzkaaaacaGLOaGaayzkaaaaleaacqWGPbqAcqGH9aqpcqaIXaqmcqGGSaalcuWGWbaCgaqbamaaBaaameaacqWGPbqAaeqaaSGaeyiyIKRaeGimaadabaGaem4AaSganiabggHiLdaaaa@469F@. Edge weights were set to logarithm of the reliability estimation of the corresponding interactions. The sequence similarity score, *h*(*q*_*i*_*, j*), between query node q_*i*_and vertex *j∈ V *was set to logarithm of the BLAST E-value between the corresponding proteins, normalized by the maximum score over all pairs.

### Pathway search module

The goal of the algorithm is to identify a matched pathway with distinct vertices yielding an optimal alignment to the query. To this end, we adapt the color coding technique of Alon et al. [41] , which serves to find simple paths (i.e., paths with distinct vertices) of a fixed length *k *in a graph. In color coding, one assigns a randomly chosen color from *{1,...,k} *to every vertex in the graph, transforming the problem of finding a simple length-*k *path to that of finding a path of length *k *that spans distinct colors. Since any particular path may be assigned non-distinct colors and, hence, fail to be discovered, many random coloring trials are executed. Below, we describe one iteration of color coding tailored to the query case.

Our algorithm starts by assigning every vertex *v *∈ *V *a color *c*(*v*) drawn uniformly at random from the set *C *=*{*1,...,*k *+ *N*_*ins*_*}*. For a given coloring, we use dynamic programming to find an optimal matching pathway. We let *W*(*i, j, S, θ*_*del*_) denote the maximum weight of an alignment for the first *i *nodes in the query that ends at vertex *j∈ V*, induces *θ*_*del *_deletions, and visits a vertex of each color in *S*. *W*(*i, j, S, θ*_*del*_) is computed recursively as follows:

W(i,j,S,θdel)=max⁡m∈V{W(i−1,m,S−c(j),θdel)+w(m,j)+h(qi,j)(m,j)∈EW(i,m,S−c(j),θdel)+w(m,j)(m,j)∈EW(i−1,j,S,θdel−1)θdel≤Ndel
 MathType@MTEF@5@5@+=feaafiart1ev1aaatCvAUfKttLearuWrP9MDH5MBPbIqV92AaeXatLxBI9gBaebbnrfifHhDYfgasaacH8akY=wiFfYdH8Gipec8Eeeu0xXdbba9frFj0=OqFfea0dXdd9vqai=hGuQ8kuc9pgc9s8qqaq=dirpe0xb9q8qiLsFr0=vr0=vr0dc8meaabaqaciaacaGaaeqabaqabeGadaaakeaacqWGxbWvdaqadaqaaiabdMgaPjabcYcaSiabdQgaQjabcYcaSiabdofatjabcYcaSiabeI7aXnaaBaaaleaacqqGKbazcqqGLbqzcqqGSbaBaeqaaaGccaGLOaGaayzkaaGaeyypa0ZaaCbeaeaacyGGTbqBcqGGHbqycqGG4baEaSqaaiabd2gaTjabgIGiolabdAfawbqabaGcdaGabaqaauaabeqadiaaaeaacqWGxbWvdaqadaqaaiabdMgaPjabgkHiTiabigdaXiabcYcaSiabd2gaTjabcYcaSiabdofatjabgkHiTiabdogaJnaabmaabaGaemOAaOgacaGLOaGaayzkaaGaeiilaWIaeqiUde3aaSbaaSqaaiabbsgaKjabbwgaLjabbYgaSbqabaaakiaawIcacaGLPaaacqGHRaWkcqWG3bWDdaqadaqaaiabd2gaTjabcYcaSiabdQgaQbGaayjkaiaawMcaaiabgUcaRiabdIgaOnaabmaabaGaemyCae3aaSbaaSqaaiabdMgaPbqabaGccqGGSaalcqWGQbGAaiaawIcacaGLPaaaaeaadaqadaqaaiabd2gaTjabcYcaSiabdQgaQbGaayjkaiaawMcaaiabgIGiolabdweafbqaaiabdEfaxnaabmaabaGaemyAaKMaeiilaWIaemyBa0MaeiilaWIaem4uamLaeyOeI0Iaem4yam2aaeWaaeaacqWGQbGAaiaawIcacaGLPaaacqGGSaalcqaH4oqCdaWgaaWcbaGaeeizaqMaeeyzauMaeeiBaWgabeaaaOGaayjkaiaawMcaaiabgUcaRiabdEha3naabmaabaGaemyBa0MaeiilaWIaemOAaOgacaGLOaGaayzkaaaabaWaaeWaaeaacqWGTbqBcqGGSaalcqWGQbGAaiaawIcacaGLPaaacqGHiiIZcqWGfbqraeaacqWGxbWvdaqadaqaaiabdMgaPjabgkHiTiabigdaXiabcYcaSiabdQgaQjabcYcaSiabdofatjabcYcaSiabeI7aXnaaBaaaleaacqqGKbazcqqGLbqzcqqGSbaBaeqaaOGaeyOeI0IaeGymaedacaGLOaGaayzkaaaabaGaeqiUde3aaSbaaSqaaiabbsgaKjabbwgaLjabbYgaSbqabaGccqGHKjYOcqWGobGtdaWgaaWcbaGaemizaqMaemyzauMaemiBaWgabeaaaaaakiaawUhaaaaa@B894@

The maximum weight of an alignment is max⁡j∈V,S⊆C,θ≤Ndel
 MathType@MTEF@5@5@+=feaafiart1ev1aaatCvAUfKttLearuWrP9MDH5MBPbIqV92AaeXatLxBI9gBaebbnrfifHhDYfgasaacH8akY=wiFfYdH8Gipec8Eeeu0xXdbba9frFj0=OqFfea0dXdd9vqai=hGuQ8kuc9pgc9s8qqaq=dirpe0xb9q8qiLsFr0=vr0=vr0dc8meaabaqaciaacaGaaeqabaqabeGadaaakeaadaWfqaqaaiGbc2gaTjabcggaHjabcIha4bWcbaGaemOAaOMaeyicI4SaemOvayLaeiilaWIaem4uamLaeyOHI0Saem4qamKaeiilaWIaeqiUdeNaeyizImQaemOta40aaSbaaWqaaiabdsgaKjabdwgaLjabdYgaSbqabaaaleqaaaaa@43ED@, *W *(*k, j, S*, θ), and the corresponding alignment is obtained through standard dynamic programming backtracking. In fact, the algorithm outputs not only the optimal match but a set of high scoring matches for each combination of number of insertions and deletions employed. The running time of each trial depends on the length of the query, the size of the network and the number of insertions and deletions allowed, and is 2^*O*(*k*+*Nins*)^*mN*_*del*_. The probability that any given path is assigned *k *distinct colors is at least *e*^-*k*-*Nins*^. Thus, for any ε ∈ (0,1), the running time of the algorithm for obtaining the optimal match with probability at least 1-ε is *ln(n/ε)2*^*O*(*k*+*Nins*)^*mN*_*del*_. We used ε = 0.01 for all runs of the algorithm, yielding a practical time of a few minutes per query (Supp.1 Table 3). The resulting pathways were filtered to remove pathways that overlap by at least 20% of their proteins.

To search a network for pathways with high interaction scores, regardless of a specific query, we ran the algorithm with a dummy path query, consisting of dummy proteins that were defined to have the same sequence similarity score with respect to all network vertices. To search a network for random pathways, regardless of their interaction score, we assigned an equal interaction score for all interactions.

### Pathway scoring module

We assigned protein pathways a functional significance score that represent their tendency to be functionally enriched given four parameters characterizing each pathway: a normalized sequence score, a normalized interaction score, number of insertions, and number of deletions. Given a set of matched pathways, logistic regression [42] was used to predict their functional enrichment based on these parameters alone. To avoid over-fitting, the set of pathways was partitioned into five equal parts. For each part, we trained the logistic regression on the remaining four parts, and used the inferred parameters to derive the scores of the pathways in the left-out part.

### Functional enrichment

Functional enrichments of protein pathways were computed based on GO process annotations [43] for their proteins. Yeast GO annotations were obtained from SGD [44] , and fly GO annotations were obtained from FlyBase [45]. For a given pathway *P *and a given term *t*, the functional enrichment score was computed as follows: suppose *P *has let *n(t) *proteins that are annotated with term *t *(or with a more specific term). Let *p(t) *be the hypergeometric probability for observing *n(t) *or more proteins annotated with term *t *in a protein subset of size *|P|*. Having found a term *t*_0 _with minimal probability *p(t*_0_*)*, the score was set to the *p*-value of the enrichment under term *t*_0_, computed by comparing *p(t*_0_*) *with the analogous probabilities for 10,000 random sets of proteins of size *|P|*.

### Expression coherency

Expression coherency of a pathway was measured as the mean absolute value of the pairwise Pearson correlations between the expression patterns of the genes that code for the pathway's proteins. To assess the significance of the expression coherency of a set of pathways, we compared it to the expression coherency distribution of a random set of pathways with the same size distribution. Gene expression measurements were obtained from Stanford microarray database [46] and included 973 and 170 conditions for yeast and fly, respectively.

## Authors' contributions

TS performed the computational analysis. DS performed the biological analysis. All authors participated in designing the study and preparing the manuscript.

## Supplementary Material

Additional File 1 supplementary figures and tables. The file contains supplementary figures 6 and 7, and tables 2 and 3. Click here for file

## Figures and Tables

**Table 1:** Functional significance of yeast and fly pathways. Functional enrichment and expression coherency of high interaction score pathways and random pathways in the PPI networks of yeast and fly.

	High interaction score pathways			Random pathways	
	Number of pathways	Functional enrichment	Expression coherency (*p*-value)	Functional enrichment	Expression coherency (*p*-value)
Yeast	271	80%	< 1e-300	17%	4.0e-4
Fly	193	20%	0.024	0%	> 0.05
