# Supplementary material for: QPath: a method for querying pathways in a protein-protein interaction network
Source: BMC Bioinformatics. 2006 Apr 10;7:199. doi: 10.1186/1471-2105-7-199 (PMC1458361; doi:10.1186/1471-2105-7-199)
Supplement: Additional File 1 — supplementary figures and tables. The file contains supplementary figures 6 and 7, and tables 2 and 3. [file 1471-2105-7-199-S1.doc]

**Supporting Information**

**Figure 6.** Yeast filamentous growth pathway query in the PPI network of yeast.


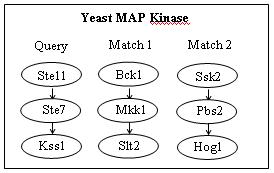


**Figure 7.** Cumulative distribution of functional enrichment p-values for (a) yeast pathways and (b) fly pathways. x-axis: Functional enrichment p-value. y-axis: Percent of pathways with *p*-value x or better.

(a)


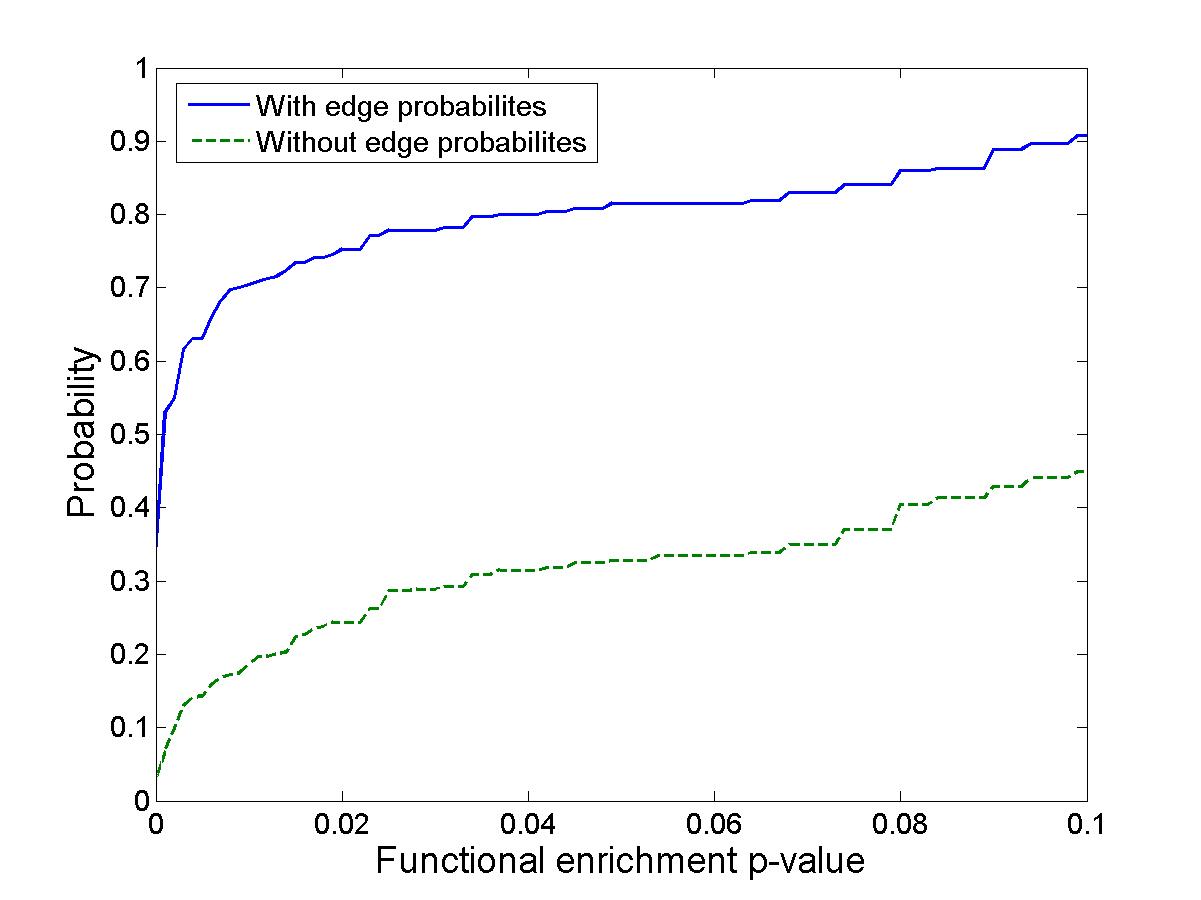


(b)


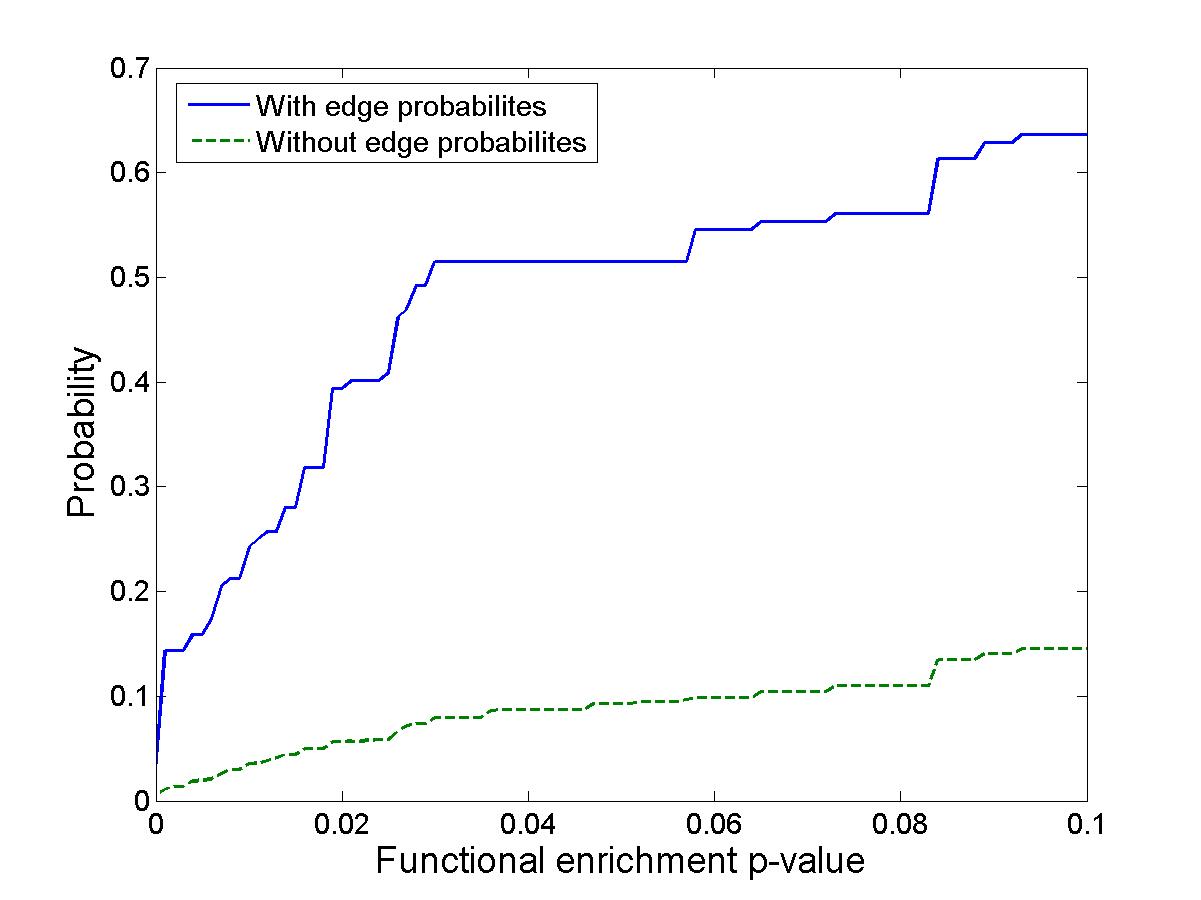


**Table 2.** (a) The fraction of yeast queries with identifiable matching fly pathway out of all yeast queries within different indel categories. (b) The fraction of matched fly pathways that are functionally enriched out of all matched fly pathways in each indel category. Indel categories not covered by any matched pathway were marked as having 0% functionally enriched pathways.

(a)

|  | 0 insertions | 1 insertions | 2 insertions | 3 insertions |
| --- | --- | --- | --- | --- |
| 0 deletions | 0 | 0 | 0 | 0.0074 |
| 1 deletions | 0 | 0.0148 | 0.0443 | 0.0701 |
| 2 deletions | 0.0185 | 0.0554 | 0.1328 | 0.2694 |
| 3 deletions | 0.0996 | 0.2325 | 0.4428 | 0.6310 |

(b)

|  | 0 insertions | 1 insertions | 2 insertions | 3 insertions |
| --- | --- | --- | --- | --- |
| 0 deletions | 0 | 0 | 0 | 1 |
| 1 deletions | 0 | 1 | 0.8333 | 0.7308 |
| 2 deletions | 0.8000 | 0.7333 | 0.6111 | 0.5890 |
| 3 deletions | 0.5185 | 0.5397 | 0.5417 | 0.5965 |

**Table 3.** The running time of the QPath algorithm (in seconds) applied to finding pathways of different lengths with maximal scores in the PPI network of yeast and fly (2nd and 3rd columns), and for querying the PPI network of fly with yeast queries (4th column). For the yeast queries in fly we show the average running time over 100 different path queries. All such queries allow up to 3 insertions and 3 deletions. In all cases, QPath used a number of color-coding iterations that is high enough to ensure optimal solutions with a probability of at least 0.99.

| Path length | Yeast | Fly | Yeast queries in fly |
| --- | --- | --- | --- |
| 4 | 3 | 3 | 37 |
| 5 | 12 | 28 | 60 |
| 6 | 44 | 89 | 136 |
| 7 | 195 | 375 | 480 |
